# Generation and Function of Non-cell-bound CD73 in Inflammation

**DOI:** 10.3389/fimmu.2019.01729

**Published:** 2019-07-26

**Authors:** Enja Schneider, Anne Rissiek, Riekje Winzer, Berta Puig, Björn Rissiek, Friedrich Haag, Hans-Willi Mittrücker, Tim Magnus, Eva Tolosa

**Affiliations:** ^1^Department of Immunology, University Medical Center Hamburg-Eppendorf, Hamburg, Germany; ^2^Department of Neurology, University Medical Center Hamburg-Eppendorf, Hamburg, Germany

**Keywords:** soluble CD73, shedding, extracellular vesicles, adenosine, immune regulation

## Abstract

Extracellular adenine nucleotides participate in cell-to-cell communication and modulate the immune response. The concerted action of ectonucleotidases CD39 and CD73 plays a major role in the local production of anti-inflammatory adenosine, but both ectonucleotidases are rarely co-expressed by human T cells. The expression of CD39 on T cells increases upon T cell activation and is high at sites of inflammation. CD73, in contrast, disappears from the cellular membrane after activation. The possibility that CD73 could act in *trans* would resolve the conundrum of both enzymes being co-expressed for the degradation of ATP and the generation of adenosine. An enzymatically active soluble form of CD73 has been reported, and AMPase activity has been detected in body fluids of patients with inflammation and cancer. It is not yet clear how CD73, a glycosylphosphatidylinositol (GPI)-anchored protein, is released from the cell membrane, but plausible mechanisms include cleavage by metalloproteinases and shedding mediated by cell-associated phospholipases. Importantly, like many other GPI-anchored proteins, CD73 at the cell membrane is preferentially localized in detergent-resistant domains or lipid rafts, which often contribute to extracellular vesicles (EVs). Indeed, CD73-containing vesicles of different size and origin and with immunomodulatory function have been found in the tumor microenvironment. The occurrence of CD73 as non-cell-bound molecule widens the range of action of this enzyme at sites of inflammation. In this review, we will discuss the generation of non-cell-bound CD73 and its physiological role in inflammation.

## Introduction

**Figure 1:**
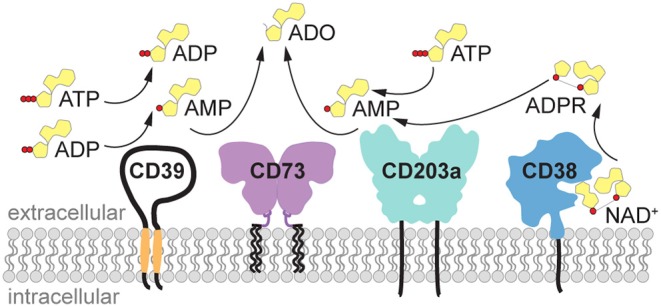
Generation of extracellular adenosine by purinergic ectoenzymes. Extracellular ATP is stepwise dephosphorylated by the ectonucleotidase CD39 (ENTPDase1) to produce ADP and AMP. Alternatively, AMP can be generated from NAD^+^ by the coordinated activity of the ecto-NAD-glycohydrolase CD38, which metabolizes NAD^+^ to ADPR, and the pyrophosphatase/phosphodiesterase CD203a (ENPP1), responsible for the conversion of ADPR to AMP. ENPP1/CD203a is also capable of generating AMP directly from ATP. Finally, the hydrolysis of AMP by ecto-5′-nucleotidase (NT5E) CD73 yields adenosine (ADO). ADO, adenosine; ADPR, adenosine diphosphate ribose; ATP, adenosine triphosphate; ADP, adenosine diphosphate; AMP, adenosine monophosphate; ENPP1, ectonucleotide pyrophosphatase/phosphodiesterase 1 (CD203a, PC-1); ENTPDase1, ectonucleoside triphosphate diphosphohydrolase 1 (CD39); NAD^+^, nicotinamide adenine dinucleotide; NT5E, ecto-5′-nucleotidase (CD73).

Under physiological conditions, the concentration of the purine nucleotides adenosine triphosphate (ATP), ADP and AMP in biological fluids and extracellular space is low (30–100 nM), while the intracellular concentration of ATP is in the millimolar range. Upon cell activation and tissue damage, ATP is readily released from the cells, resulting in a surge of pericellular ATP (1–3). Indeed, the concentration of ATP measured in close proximity to the cell using imaging methods reached 60 μM a few minutes after T cell stimulation (4), and this concentration is sufficient to stimulate P2 receptors and elicit pro-inflammatory signals. The excess of extracellular ATP is rapidly hydrolyzed by ectonucleotidases such as CD39 or ectonucleotide pyrophosphatase/phosphodiesterases (i.e., ENPP1, also known as CD203a or PC-1) to generate ADP and finally AMP, the substrate for the ecto-5′-nucleotidase CD73 (NT5E) (Figure 1). AMP is converted to adenosine primarily by CD73 and, less efficiently, by alkaline phosphatase. AMP can likewise be generated from extracellular nicotinamide adenine dinucleotide (NAD^+^) by the coordinated activity of the ecto-NAD-glycohydrolase CD38, which metabolizes NAD^+^ to ADPR, and the pyrophosphatase/phosphodiesterase CD203a (ENPP1), responsible for the conversion of ADPR to AMP (5, 6) (Figure 1). ENPP1 can also convert ATP directly to AMP. Similar to ATP, the concentration of NAD^+^ is higher inside the cells (high micromolar to millimolar range) than in the extracellular space, where it has been measured at 20–60 nM (7), and is released upon cell damage.

Adenosine, by binding to P1 receptors on immune cells elicits predominantly anti-inflammatory signals (8). As the rate-limiting enzyme for adenosine production, CD73 plays a key role in the balance between inflammation and immune suppression (9, 10). Independently of its enzymatic activity, CD73 has been reported to provide a costimulatory signal for lymphocyte activation (11, 12), and mediate lymphocyte adhesion to the endothelium (13). More recent data, however, suggest that CD73-mediated production of adenosine and subsequent signaling through adenosine receptors restricts leukocyte adhesion to endothelium (14–16). Surprisingly, CD73-deficiency protected animals from experimental autoimmune encephalomyelitis by reducing migration of pathogenic immune cells into the brain (17), but resulted in larger cerebral infarct volumes and increased local leukocyte infiltration in a model of stroke (18). These apparently contradictory results on the role of CD73 in brain inflammation might be explained by adenosine availability and differential adenosine receptor engagement in chronic and acute settings, underscoring the multifaceted role of CD73 and adenosine in inflammation (19). Expression of CD73 and adenosine signaling have also been involved in the regulation of vascular permeability by promoting endothelial barrier function (20).

While the expression of CD73 on endothelia has been documented across species, the expression of CD73 on immune cells is species-specific. In humans, CD73 is expressed on most B cells (certainly on all mature naïve B cells) and on some T cell subsets, namely naïve cells in the CD8 compartment [ImmGen database consortium Heng et al. (21)], innate-like T cells (22), and a small subset of memory CD4 T cells (23, 24). In mice, CD73 is expressed on peritoneal macrophages, most T cells, including Tregs, NK cells, and in the B cell compartment it is preferentially expressed in mature class-switched and germinal center B cells (25–28). Remarkably, CD39 and CD73 are rarely co-expressed on human conventional T cells in the periphery, and the expression of CD73 is a seldom event on human Tregs (29–31), even though murine Tregs do express these ectoenzymes constitutively (32). In the inflamed joints of patients with arthritis CD39 is upregulated on T cells, while the expression of CD73 is low (25, 29, 33, 34). Accordingly, *in vitro* activation of human conventional T cells results in the upregulation of CD39 and loss of CD73 from the cell membrane (11, 25). Interestingly, CD39 and CD73 are co-expressed in a subset of Th17 cells with suppressive features (24, 35). These cells are prominent in the lamina propria, and were found decreased in patients with inflammatory bowel disease, underscoring their relevance for the control of inflammation in the gut (24).

The enzymatic activities of CD39 and CD73 are complementary for the generation of adenosine and subsequent control of the inflammatory response, but since their co-expression in T cells is rare, it is plausible that the AMPase activity of CD73 is provided in *trans* by neighboring cells of different lineages. How CD39-expressing T cells are endowed with AMPase activity in the context of inflammation is not completely understood, but we have learned from the tumor microenvironment that CD73-positive extracellular vesicles (EVs) contribute to the dampening of anti-tumor immune responses (36, 37), and EVs derived from murine regulatory T cells display AMPase activity (38). Vesicular release is facilitated upon cell activation (39) and the loss of cell surface CD73 in T cells of the synovial fluid in patients with arthritis or after *in vitro* stimulation (25, 33) suggests that CD73 may be shed from the cell surface, either as a soluble molecule or in form of vesicles. As a consequence, non-cell-bound and enzymatically active CD73 spreads at sites of inflammation and modulates the immune response by generating an adenosine-rich anti-inflammatory environment. In this review we will discuss the generation of non-cell-bound CD73 and its physiological role in inflammation.

## Evidence for Non-Cell Bound and Functionally Active CD73

CD73 is a 71 kDa homodimer attached to the plasma membrane by a GPI-anchor (40, 41). It can be found as a membrane-bound phospholipase C-sensitive form, a membrane-bound phospholipase C-resistant form, and as a soluble variant of the protein deriving from the GPI-anchored form (42, 43). Soluble and enzymatically active CD73 could be purified from the supernatant of human placental extracts, showing similar affinity for AMP as the membrane-bound form (42). Moreover, human plasma and serum (44–46) as well as vitreous fluid (47) exhibit AMPase activity that can be specifically blocked by ecto-5′-nucleotidase inhibitor adenosine 5′-(α,β-methylene)-diphosphate (APCP), indicating the presence of soluble CD73 (sCD73).

CD73 is not the only ectoenzyme that exists in soluble form in peripheral blood. Other purine-metabolizing enzymes, namely alkaline phosphatase, adenosine deaminase (ADA), CD38, ATP-degrading enzymes like ENPPs and CD39, as well as ATP-regenerating kinases are also present in human plasma (46, 48–51), indicating that there is a complex network of enzymatically active molecules shifting the balance of purinergic signaling and thereby modulating the immune response.

Shedding of CD73 can occur through hydrolysis of the GPI-anchor by endogenous phospholipases (42, 43, 52) or by proteolytic cleavage (53). Soluble CD73 from human placenta contained myo-inositol, a part of the GPI-anchor linked to the protein after phospholipase shedding, confirming release from the membrane by endogenous phospholipase C or D (42). These two phospholipases cleave the GPI-anchor at different sites, and only cleavage by phospholipase C leaves the cross-reacting determinant (CRD) epitope intact (54). Using an antibody that recognizes the CRD, this epitope was detected in purified bovine sCD73, pointing at phospholipase C-mediated shedding of CD73 (43). Partial resistance of placental and lymphocytic CD73 to phospholipase C cleavage suggested the existence of a non-GPI-linked version of CD73 (42, 55). However, none of the cloned CD73 cDNAs from different species was found to encode for a variant with a conventional membrane domain (56). Interestingly, there is evidence that the observed resistance to phospholipase C-mediated shedding is due to palmitoylation of the inositol-group (57). Therefore, palmitoylation of the GPI-anchor may represent a regulatory mechanism to control shedding of CD73. Of note, palmitoylated GPI-anchored proteins remain sensitive to phospholipase D (57, 58), and extracellular phospholipase D capable of shedding CD73 is present in mammalian plasma (59). Interestingly, alkaline phosphatase, which can also generate adenosine from AMP, is also GPI-anchored (6), indicating that phospholipase-mediated cleavage may be a common mechanism by which immune cells can prevent autocrine adenosine-mediated inhibition.

In addition to phospholipase-mediated shedding, proteolytic cleavage of CD73 can also generate a soluble form of the enzyme. Matrix metalloproteinase 9 (MMP-9) has been shown to cleave lipid raft-associated CD73 from the membrane of activated mouse retinal pigment epithelial cells. The generated sCD73, however, was enzymatically inactive (60). In contrast, an active form of sCD73 generated by proteolytic cleavage and lacking the GPI-anchor was found in bull seminal plasma (53). This soluble protein differs from the GPI-anchored form in its posttranslational modifications, aggregation patterns and in enzymatic activity, since it has a lower affinity for AMP compared to the membrane-bound version (53).

Direct comparison of AMPase activity among lymphocytic membrane-bound CD73, GPI-anchored CD73 inserted into an artificial lipid bilayer and sCD73 revealed lower catalytic efficiency of the membrane-bound forms. Further, phospholipase C-mediated release of CD73 from the membrane results in enhanced ectonucleotidase activity (61, 62). Thus, phospholipase-mediated release of CD73 from the cell membrane does not only increase its range of action, but also boosts its enzymatic activity.

## CD73 in Extracellular Vesicles

A characteristic feature of GPI-anchored proteins is that they are not evenly distributed on the cell surface, but rather enriched in specific domains, the so-called lipid rafts or detergent-resistant membranes, which serve as platforms for signal transduction (2, 63). GPI-anchored proteins, probably by the fact that they are residents of these specific domains, are also present in EVs (64). EVs are lipid bilayer vesicles released by most cell types that can transport different kinds of cargos such as proteins, lipids, mRNA, non-coding RNA and DNA (65). According to their origin, EVs can be differentiated into exosomes (with an endosomal origin) and ectosomes or microvesicles/microparticles (originated by vesicle shedding at the plasma membrane) (66), including apoptotic bodies (originated from the plasma membrane of cells undergoing apoptosis) (67) or large oncosomes (originated from the plasma membrane of cancer cells) (68, 69). Depending on their origin, EVs vary in size from 30 nm to various μm (70). CD73 protein and AMPase activity have been detected in EVs, in particular exosomes, derived from cancer cells (36, 71), regulatory T cells (38), mesenchymal stem cells (72), and also from human plasma (30). Moreover, it is unclear if the soluble CD73 (or its enzymatic activity) in human body fluids reported in other studies is truly soluble or vesicle-associated, or both, since the presence of EVs was not addressed in those studies.

Extracellular vesicles derived from different cancer cell lines co-express CD39 and CD73 and are capable of hydrolyzing ATP to adenosine, modulating the tumor microenvironment and T cell function independently of direct contact to immune cells (36). Moreover, B cell-derived CD39^+^CD73^+^ EVs are elevated in the serum of colon cancer patients and metabolize tumor-derived ATP to adenosine, impairing anti-tumor responses of CD8 T cells (37). In both studies the inhibition of CD73 had a major impact on T cell function. Vesicles isolated from plasma of healthy donors or patients with neck squamous cell carcinoma exhibited AMPase activity and converted ATP to adenosine in co-cultures with CD39^+^ Tregs, demonstrating that co-expression of CD39 and CD73 on the same cell is not necessary to endow Tregs with adenosine-mediated suppressive function (30). Thus, the enzymatic activity of CD73 in EVs contributes significantly to impair anti-tumor immune responses.

## Physiological Relevance of Non-Cell Bound CD73 in Inflammation

Murine Tregs express both CD39 and CD73, and EVs derived from activated murine Tregs are CD73-positive, convert AMP to adenosine, and mediate immune suppression (38). In contrast, activated human T cells (conventional or regulatory) express CD39, but not CD73. The analysis of the enzymatic activity responsible for ATP degradation in human blood revealed that while ATPase and ADPase activities are primarily mediated by cell-associated enzymes, it is the enzymes present in body fluids, e.g., plasma, that perform the last step and convert AMP to adenosine (44, 48, 73). Indeed, CD73-specific AMPase activity can be measured in the supernatant of synovial fluid in patients with arthritis (74, 75). In contrast to the cell-free moiety, synovial fluid T cells from patients with arthritis show high expression of CD39 and robust production of AMP, but low levels of CD73 and, consequently, poor generation of adenosine (33). Mechanistically, soluble CD73 or CD73-containing EVs locally released upon activation could provide the missing AMPase activity, thus completing the ATP degradation cascade to adenosine (30, 72, 75). Moreover, it is plausible that the AMP generated pericellulary diffuses short distances within the extracellular space from the generating cell to a CD73-expressing cell to be metabolized. Hence, production of adenosine does not necessarily require the co-expression of the ATP- and AMP-degrading enzymes in the same cell, and since the AMPase activity can be provided in *trans*, differences in CD73 expression on immune cells between humans and mice may be of limited relevance. Of note, the contribution of non-cell-bound CD38 and ENPPs for the generation of AMP is much less explored, and only recently the enzymatic activities for these enzymes have been described in EVs from multiple myeloma cells (76).

ATP and NAD^+^ are released from activated or dying cells at sites of inflammation. In parallel, activated immune cells upscale the expression of purinergic enzymes CD39 and CD38 and lose CD73 from the cell membrane, either as a soluble molecule or contained in EVs. Local stromal cells, such as endothelial or mesenchymal stem cells, can also release CD73-containing vesicles. ATP and NAD^+^ will be degraded either pericellularly by cell bound enzymes, or by soluble or vesicular enzymes to produce adenosine, which by engaging P1 receptors on immune cells will dampen the immune response. Compared to cell-associated CD73, the non-cell-bound form presents two advantages: first, it increases the distance range of enzymatic activity; second, the activated 'donor' cell that loses membrane CD73 is protected from pericellular adenosine that could terminate its effector function prematurely. Even though CD73 in soluble form is enzymatically more active than the membrane-bound variant (61), the release of membrane-bound enzymes in form of vesicles has several advantages: (i) first, extended half-life, since degradation is circumvented; (ii) improved efficiency of enzymatic activity, due to the concentration of the enzyme in microdomains; (iii) facilitation of distant transport, permitting systemic modulation; (iv) finally, the fusion of the EVs with the plasma membrane of target cells can provide them with enzymatic activities that were lacking.

The adenosinergic pathway is controlled at several checkpoints: First, the regulation of CD73 expression and shedding dictates the rate of extracellular adenosine production at the inflammation site. Importantly, higher concentrations of non-cell-bound CD73 and of its substrate AMP do not necessarily result in high extracellular adenosine, since the availability of adenosine is further modulated by its degradation to inosine mediated by adenosine deaminase, and by cellular uptake facilitated by nucleoside transporters (2, 77). Moreover, high concentrations of ATP and ADP can inhibit CD73 enzymatic activity, as it was demonstrated in the supernatants of inflamed ileum organ culture in a model of postinflammatory ileitis (77). Finally, the concentration of available adenosine will determine if the high (A_2A_) or low (A_2B_) affinity receptors are engaged in the target cells (78), and further modulation of adenosine signaling occurs in hypoxic conditions, when A_2B_ receptors are specifically upregulated (79) and silence A_2A_ receptor signaling (80).

**Figure 2:**
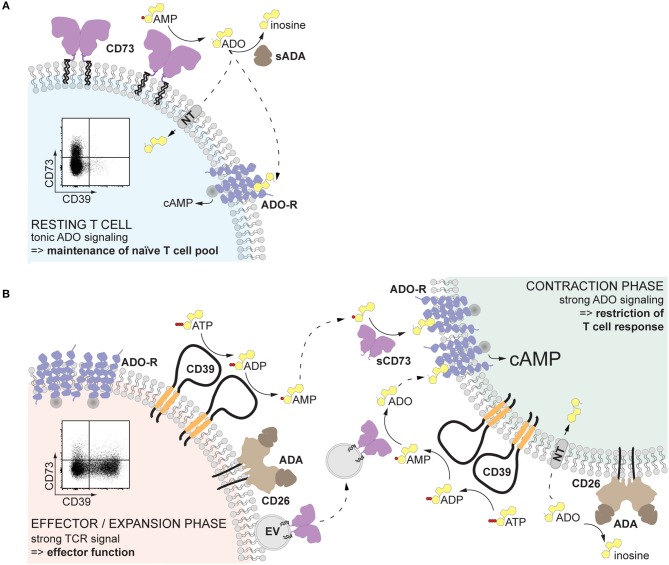
Proposed role of non-cell-bound CD73 in inflammation. **(A)** Human resting T cells, especially naïve CD8^+^ T cells, express CD73 on the cell surface. The pericellularly generated adenosine binds to adenosine receptors, providing a tonic signal that serves to maintain the naïve T cell pool. **(B)** Upon T cell receptor activation, T cells upregulate CD39, which hydrolyzes local extracellular ATP to AMP. At the same time, CD73 is released from the cell membrane and diffuses away, maintaining the local adenosine production low and therefore preserving T cell effector function (left). Soluble or vesicular CD73 can reach other activated T cells and act in a paracrine way generating adenosine. Engagement of high affinity adenosine receptors will result in elevated cAMP and restriction of the T cell response, inducing the contraction phase (right). The availability of adenosine is further regulated by the activity of adenosine deaminase, which converts adenosine into inosine, and transport into the cell by nucleoside transporters. Solid-lined arrows: enzymatic activities; dotted-lined arrows: diffusion. ADA, adenosine deaminase; ADO, adenosine; ADO-R, adenosine receptor; cAMP, cyclic AMP; NT, nucleoside transporter; sADA, soluble ADA; EV, extracellular vesicle.

When is AMPase activity desirable? In resting T cells, since tonic adenosine signaling is necessary for the maintenance of the naïve T cell pool (81) (Figure 2A). After infection or sterile inflammation, immune regulation is necessary for the contraction of the immune response and for limiting immunopathology (Figure 2B). Beyond FoxP3^+^ Treg cells, many other immune cell types can acquire regulatory properties, but too much control can lead to insufficient immune responses and subsequent pathogen expansion or impaired tumor control. Therefore, a mechanism that can be quickly modulated and acts on different cell types represents a substantial advantage. Adenosine-mediated immune suppression fulfills these requirements, since inhibitory P1 receptors are widely expressed on immune cells, and the enzymatic activities for adenosine generation and degradation are quickly modulated. The finding that the enzymes responsible for the generation of adenosine are readily shed from the cell membrane and can be transported to act on other cell types further endorse adenosine as an ideal regulator of the immune response (Figure 2). Indeed, after myocardial infarction, T cell-mediated production of adenosine was found enhanced and beneficial for recovery (82). Similarly, a transient surge of systemic adenosine is detected after stroke in humans (83), and high AMPase activity can be measured in the plasma of neonates, probably ensuring tolerance to the new microbial environment (44). Understanding how systemic AMPase activity is regulated will provide clues for therapeutic intervention. While in cancer immunotherapy the blockade of CD73 seems a promising strategy (84), the systemic usage of CD73 to dampen inflammation is hampered by vasodilation and subsequent decrease in blood pressure in response to adenosine receptor engagement in the vasculature. Therefore, strategies that restrict adenosine signaling to the site of inflammation must be arranged, as elegantly shown by Flögel et al. (85) in a model of arthritis. The use of locally delivered CD73-containing EVs may provide a further therapeutic option.

## Author Contributions

ES, AR, and ET conceived the structure of the review and wrote the manuscript. RW and BR contributed with the part related to shedding of CD73. BP and TM contributed with the part on extracellular vesicles. H-WM and FH revised and corrected the manuscript. All authors read the final version of the manuscript and approved it for submission.

### Conflict of Interest Statement

The authors declare that the research was conducted in the absence of any commercial or financial relationships that could be construed as a potential conflict of interest.
